# Evaluating Sex‐Based Disparities in STEMI Care: A Call for Methodological Rigor

**DOI:** 10.1002/clc.70298

**Published:** 2026-04-11

**Authors:** Noor‐ul‐Eman Haider

**Affiliations:** ^1^ Khyber Medical College Peshawar Pakistan

1

Dear Esteemed Editor,

We read with much interest the paper by Evelo et al., “Evaluation of Sex‐Based Differences in the Prescription of the Combination of Evidence‐Based Medicine After the Occurrence of an Acute ST‐Elevation Myocardial Infarction,” published in Clinical Cardiology [1]. The authors are to be commended on using electronic health records to investigate an intriguing question: whether women and men experience similar prescriptions of evidence‐based treatments following STEMI. Their paper is relevant, especially with the recent fervor regarding sex differences in cardiovascular treatment. Two telling methodological concerns render the quality of the findings and, in our view, warrant particular attention prior to reaching conclusions.

One of them quite critically is omitting to control confounding when presenting clinical outcomes. The authors have stated that “all mentioned secondary outcomes were not adjusted for confounders.” The paper does note, though, that the women were much more likely to have adverse events, with 6‐month mortality of 5.0% versus 2.7% in men, and 6‐month stroke rate of 5.5% versus 2.9% in men. Such rates, unadjusted as they are multivariable, are vulnerable to impugning causative association with the adverse outcome of sex per se [2]. In the real world, such heterogeneity may represent heterogeneity in procedural volume, comorbidity burden, age, or frailty—components the authors knew were present but did not consider in outcome analyses [3]. Without control, readers cannot help but wonder whether under‐treatment of interventions was somehow mediating observed heterogeneity, or whether they are risk‐adjusted at baseline. Additional research with multivariable Cox regression, propensity score matching, or mediation analysis would provide more reliable data [4] on whether varying prescribing is responsible for poorer outcomes in women.

Secondly, loss of significant clinical covariates owing to missing data is troubling. The authors state that “variables were omitted from analysis if more than 50% of the values were missing.” Yet even those factors short of threshold were recorded as missing at very high rates. Notably, left ventricular ejection fraction (**LVEF**)—a critical determinant of treatment eligibility and outcome [5, 6, 7]—was “unknown” in 44.8% of women and 40.7% of men. Clustering nearly half the cohort as “unknown” could obscure clinically important differences and ruin adjusted models. Practice in clinical epidemiology now advises the application of multiple imputation with auxiliary variables to mitigate such missingness bias [8, 9]. Without robust control, multivariable estimates of this study—for example, the aforementioned adjusted odds ratio for sex (**1.01; 95% CI: 0.73–1.39**)—are at risk of instability and misattribution.

These frailties all argue for more methodological gravitas in large EHR studies. By controlling confounding in comparisons between adverse outcomes and ascribing the extreme level of missingness of LVEF, authors would be in a position to significantly enhance validity of results and establish if sex differences still persist in STEMI management. Given that this report is polemic in raising a fundamental clinical and policy question, we urge reconsideration on these grounds with the added benefit of solidifying the evidence base and the methodology precedent for future research into cardiovascular outcomes.

## Data Availability

Data sharing is not applicable to this article, as no new data were created or analyzed in this study.
